# Mid-to Long-term Outcomes After Single Radiofrequency Ablation of Nontoxic and Toxic Thyroid Nodules

**DOI:** 10.1210/clinem/dgaf611

**Published:** 2025-11-04

**Authors:** Harald Dobnig, Karin Amrein

**Affiliations:** Thyroid Practice and Clinic for Radiofrequency Ablation and Minimally Invasive Interventions, Kumberg 8062, Austria; Department of Internal Medicine, Division of Endocrinology and Diabetology, Medical University of Graz, Graz 8010, Austria

**Keywords:** RFA, thyroid nodules, autonomous adenoma, thermoablation

## Abstract

**Objective:**

To evaluate the mid- to long-term recurrence rates after a single radiofrequency ablation (RFA) of benign thyroid adenomas.

**Design:**

Retrospective review of data collected in a standardized prospective database.

**Setting:**

Single-center thyroid clinic in Austria.

**Participants:**

Eight hundred ninety-six consecutive patients with either nontoxic nodules (n = 765) or toxic adenomas (n = 131).

**Intervention:**

Mono- or bipolar RFA under local anesthesia.

**Main Outcome Measures:**

Recurrence defined as increase in nodule size after RFA treatment by ≥50% from its smallest volume (nontoxic adenomas) or relapse of hyperthyroidism (toxic adenomas).

**Results:**

Follow-up periods of 3 years or more were available for 437 nontoxic and 58 toxic adenomas. The median baseline volume was 10.1 mL [interquartile range (IQR) 5.5-20] for nontoxic and 7.2 mL (IQR 4.2-14.3) for toxic nodules. Solid or predominantly solid nodules comprised 81% and 93%, respectively. After 1, 3, and 5 years, the average nodule volume decreased by 79%, 82%, and 86% for nontoxic and by 84%, 88%, and 89% for toxic nodules, remaining stable afterwards.

In 54 patients (7.1%) with nontoxic nodules, regrowth occurred, with a cumulative incidence rate of 17.3% over a maximum of 8 years. Among 15 patients (11.5%) with toxic adenomas, recurrence of hyperthyroidism was observed over 7 years (21.6% cumulative incidence). Fourteen of these patients became euthyroid after low-dose radioiodine treatment (n = 11) or a second RFA (n = 3). Documented complications included 2.3% moderate and 0.3% severe cases.

**Conclusion:**

In our cohort, a single RFA treatment resulted in favorable mid- to long-term outcomes in 83% of nontoxic and 78% of toxic adenoma patients, with an acceptable complication rate.

Long-term studies of patients treated with a single radiofrequency ablation (RFA) are crucial for cost-benefit analyses and for informing patients about potential risks and benefits. However, these studies are in general costly and challenging since many patients who feel better are hard to convince to return for follow-ups. Additionally, centers offering such treatments are still often far from where patients live, complicating long-term outcome documentation. Sim et al reviewed multiple studies and concluded that a 3-year period is suitable to define “long-term” follow-up for benign thyroid nodules treated with thermoablation (1).

Such treatment studies have complex variables, making firm conclusions difficult. Many studies report that patients needed multiple RFA treatments due to persistent symptoms or viable nodule tissue (2-7). Some long-term studies had small sample sizes (2, 4-7) or low initial mean nodule volumes (8, 9). Nodule regrowth was sometimes omitted (2) or defined as exceeding its initial size (10). However, the definition of recurrence, where the nodule size increases by ≥50% from its smallest volume, is now widely accepted (11).

When the number of measurements is unequal due to dropouts, it is critical to use an incidence rate for regrowth, which was not consistently applied (4).

When RFA was introduced in Austria in 2014, health insurance covered only about 10% of the treatment costs. Since 2022, the situation has improved (12), but multiple treatments still pose financial concerns. Reviewing data from countries without a long tradition in ablation procedures such as Korea, China, or Italy also seems crucial.

The primary objective of this study thus was to document recurrences in patients with nontoxic and toxic adenomas following a single RFA treatment. Additionally, the study aimed to describe long-term reductions in nodule volume and improvements in clinical symptoms.

## Materials and Methods

This analysis reviews a prospectively designed database from April 2014 to July 2024. Data from all consecutively treated patients, including previous relevant findings; technical aspects of the RFA procedures; and follow-up visits were systematically recorded in an Excel database starting from the first patient.

### Patients

The inclusion criteria of this study were as follows: (1) all patients in this study had cytologically confirmed benign nodules (Bethesda II) and unremarkable cervical lymph node status. For nonspongiform nodules, cytology was repeated. Fine-needle capillary cytology was not deemed necessary for patients with toxic adenomas. (2) Calcitonin levels were required to be within the reference range. (3) The nodules had to cause pressure symptoms and/or cosmetic problems. Patients were excluded if (1) cytology was other than Bethesda 2, (2) they had giant adenomas requiring palliative care, (3) nodules were in large thyroids with disseminated autonomy scheduled for radioiodine treatment after RFA, (4) they were lost to follow-up, (5) the nodule was not of thyroid origin, (6) postinterventional infection required surgery, (7) RFA was aborted due to a panic attack, or (8) they had parathyroid adenoma.

At the initial consultation, all patients had thyroid scintigraphy or provided diagnostic imaging, allowing adenomas to be classified as “toxic” or “nontoxic” based on lab results. Hyperthyroid patients had TSH values ≤0.4 mU/L, or thyrostatic medications had already been initiated.

The majority of patients (60.1%) sought treatment after internet research, while 15.5% and 11.1% were referred by a thyroid specialist or medical specialist/general practitioner, respectively. A recommendation from another RFA patient accounted for 2.9%, and 10.4% were recruited from our own patient cohort.

This study adhered to the Declaration of Helsinki guidelines. All participants signed a comprehensive 7-page patient information and consent form, consenting to data analysis and publication. The protocol for this retrospective analysis was approved by the Ethics Committee of the Province of Styria (ABT08-72958/2024-5). One-year results from 277 patients were included in a previously published study (13).

### Clinical Evaluation and Follow-up

Following a consensus statement (14), symptom and cosmetic scores were recorded at baseline and each follow-up examination. Check-ups occurred after 3 months, 1 year, and then annually until December 2024. At each visit, thyroid and nodule volumes were measured, and lab tests (TSH, free T3, free T4, optional thyroglobulin antibodies, thyroid peroxidase antibodies, thyrotropin receptor antibodies) were performed. Nodule shrinkage was calculated with volume reduction rate (VRR; percent) = (V1 – V2)/V1 × 100, where V1 and V2 are initial and follow-up volumes in mL. Nine patients (1.2%) with nontoxic nodules and 2 patients (1.5%) with toxic nodules had a second RFA, showing no recurrence.

### Regrowth

For nontoxic nodules, this study used the standard international definition of regrowth, which is an increase in residual nodule volume by more than 50% compared to the lowest postinterventional nodule volume (11). In patients with toxic nodules, a recurrence was defined as a drop in the TSH value to 0.4 mU/L or less independent of changes in nodule volume. Data from follow-up visits in which patients were censored due to “regrowth” or “relapse of hyperthyroidism” were included but excluded from subsequent analyses.

### Complications and Side Effects

The recording of mild, moderate, and severe complications and side effects was based on the Society of Interventional Radiology Reporting Standards (15). Pain during RFA treatment was not recorded as a complication or side effect and could typically be managed by a second or third lidocaine injection and did not necessitate discontinuation of treatment. Temporary vocal cord paralysis caused by lidocaine as well as small hematomas were also not classified as side effects, whereas significant hematomas were.

### RFA Intervention

Technical details of the RFA intervention are thoroughly described in previous publications (13, 16). Monopolar, internally cooled 18G electrodes and a VIVA RF generator (STARmed, Seoul, Korea) or a bipolar ablation system (CelonLab POWER, Olympus, Hamburg, Germany) were used. All RFA procedures were carried out by 1 operator (H.D.), who had 24 years of thyroid ultrasound experience but no prior interventional qualifications at the study's outset. Treatments were outpatient procedures utilizing the transisthmic moving-shot or multiple overlapping shot technique with subcutaneous and pericapsular lidocaine infiltration. Sedation was not used in any of the cases. RFA was terminated as soon as the entire nodule appeared clearly hypoechogenic and no positive Doppler signal within the treated nodule was detectable.

After publication, techniques such as artery-first and marginal venous ablation (17) and hydrodissection (18) were gradually adopted and became standard practice. Since 2020, an ice-cold 5% dextrose injection rescue maneuver has been used for voice abnormalities during RFA treatment (19). Nodule composition was defined as follows: solid (percent cystic portion: ≤ 10%), predominantly solid (11-50%), predominantly cystic (51-90%), and cystic (≥90%).

### Internet Survey

Within 12 months of the RFA, surveys on patient information, tension, pain, and overall satisfaction were sent to 360 patients using SurveyMonkey and analyzed in August 2018. An additional set of identical questions was distributed to 178 more patients in August 2019. As these analyses produced virtually identical results, no further surveys were conducted.

### Statistical Analysis

Continuous variables are shown as mean ± SD, while nonnormally distributed variables are presented as median with interquartile range (IQR). Differences between patients with and without regrowth or in delivered energy per ml of nodule volume (nontoxic vs toxic adenoma groups) were analyzed using the Mann–Whitney *U*-Test. The chi-square test evaluated differences in Symptom and Cosmetic scores within a patient group or differences in complication rates between time periods. The Kaplan–Meier method assessed the cumulative incidence of regrowth or hyperthyroidism relapse. Cox regression and multiple linear regression identified independent predictors of regrowth, VRR, or hyperthyroidism relapse. Statistical significance was set at *P* < .05, and all analyses used SPSS version 30.0 (SPSS Inc., Chicago, IL, USA).

## Results

### Characteristics of Patients, Nodules, and RFA

**Figure 1.**
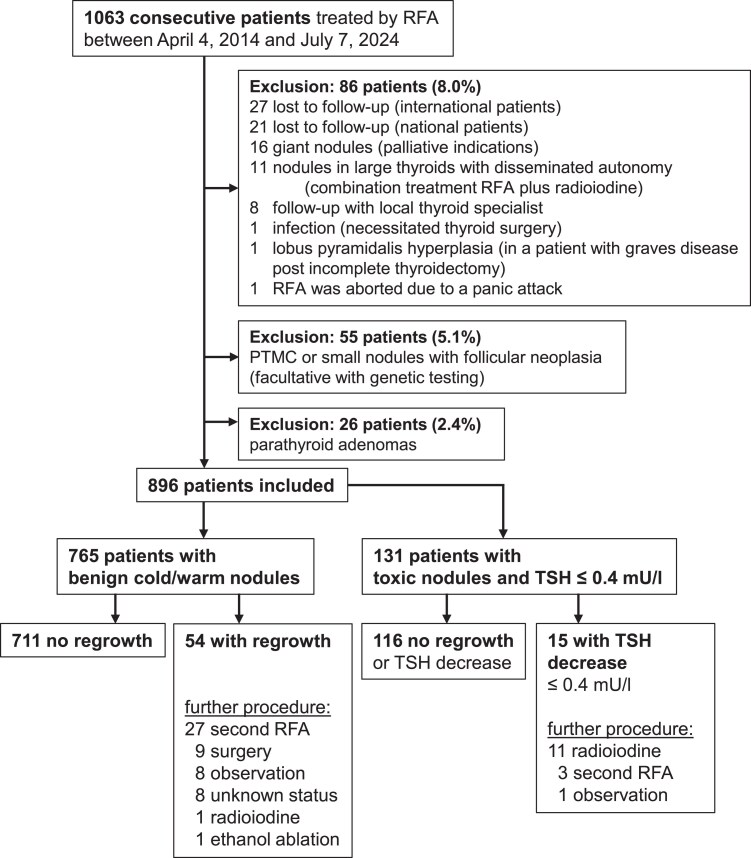
Patient flowchart. Abbreviations: PTMC, papillary thyroid microcarcinoma; RFA, radiofrequency ablation.

Table 1 shows data on patient and nodule characteristics for nontoxic and toxic adenomas and RFA treatment specifications. The patient flowchart is illustrated in Fig. 1. A total of 765 patients with 883 nontoxic nodules and 131 patients with 144 toxic adenomas were treated. When patients first came to our institution, 69% with nontoxic nodules already had a recommendation for surgery, and 73% of patients with toxic adenomas had a recommendation for radioiodine therapy or surgery. The initial median nodule volume was 10.1 mL (IQR 5.5-20) for nontoxic and 7.2 mL (IQR 4.2-14.3) for toxic adenomas. Solid or predominantly solid nodules accounted for 80.6% of nontoxic and 92.9% of toxic adenomas. Monopolar ablation was used in 76.5% of nontoxic nodules and 86.8% of toxic adenomas; others received bipolar ablation.

**Table 1.** Characteristics of patients, nodules, and technical aspects

	Patients with nontoxic nodules	Patients with toxic nodules		Patients with nontoxic nodules	Patients with toxic nodules
**Patients**			**Nodules**		
Patients, n	765	131	Number of treated nodules, n	883	144
Female/male, n (%)	566 (74)/199 (26)	97 (74)/34(26)	Baseline nodule volume, mL, median (IQR)	10.1 (5.5-20)	7.2 (4.2-14.3)
Age, years (mean ± SD)	52 ± 13	51 ± 14	Maximum nodule diameter, cm, median (IQR)	3.6 (3.0-4.5)	3.3 (2.7-4.2)
Patients with single RFA session, n (%)	756 (98.8)	129 (98.4)	Number of nodules treated per patient, median (range)	1 (1-5)	1 (1-6)
Patients with 2 RFA sessions, n (%)	9 (1.2)	2 (1.5)	Multinodular toxic adenomas, n (%)	NA	13 (9.9)
Bilateral RFA, %	11.4	20.1	Nodule composition,*^c^* n (%)		
Outside recommended surgery, %	69.3	NA	Solid	539 (61.7)	95 (65.9)
Outside recommended radioiodine treatment or surgery, %	NA	73.6	Predominantly solid	165 (18.9)	39 (27.0)
Cosmetic score,*^a^* % of patients			Predominantly cystic	130 (14.8)	9 (6.2)
0	14.4	32.1	Cystic	39 (4.4)	1 (0.7)
1	12.5	16.8	Thyroid volume, mL, median (IQR)	27.0 (19.5-40.5)	22.8 (17.7-30.9)
2	21.4	16.0	Lobe volume ipsilateral, mL, median (IQR)	18.9 (12.6-30.4)	15.1 (11.3-20.8)
3	51.7	35.1	Lobe volume contralateral, mL, median (IQR)	6.8 (4.8-9.8)	6.1 (4.6-9.7)
Symptom score,*^b^* VAS 1-10, %			**RFA (technical aspects)**		
≤3	56.7	55.7	Monopolar RFA, number of nodules, n (%)	583 (76.5)	125 (86.8)
4-7	35.5	37.4	Bipolar RFA, number of nodules, n (%)	179 (23.5)	19 (13.2)
8-10	7.8	6.9	Power, W, mean ± SD	50.7 ± 19.0	55.1 ± 18.3
Follow-up period, months, median (IQR)	36 (12-60)	36 (12-60)	Energy, kcal, mean ± SD	3.9 ± 3.3	3.9 ± 2.3
			Generator-time, minutes, mean ± SD	7.9 ± 5.7	7.4 ± 4.1
			Overall duration of treatment, minutes, mean ± SD	37.5 ± 12.1	37.7 ± 11.0

Abbreviations: IQR, interquartile range; NA, not applicable; RFA, radiofrequency ablation; W, watt.

^*a*^Cosmetic score: 0 = no nodule palpable, 1 = nodule palpable, 2 = nodule visible when swallowing, 3 = nodule visible with naked eye.

^*b*^For obtaining a symptom score, patients were asked the following question: To what overall degree does the nodule bother you cosmetically or in terms of functional impairment or reduced psychological well-being on a scale of 0 to 10?

^*c*^Nodule composition: solid (percent cystic portion: ≤ 10%), predominantly solid (11-50%), predominantly cystic (51-90%), cystic (>90%).

**Figure 2.**
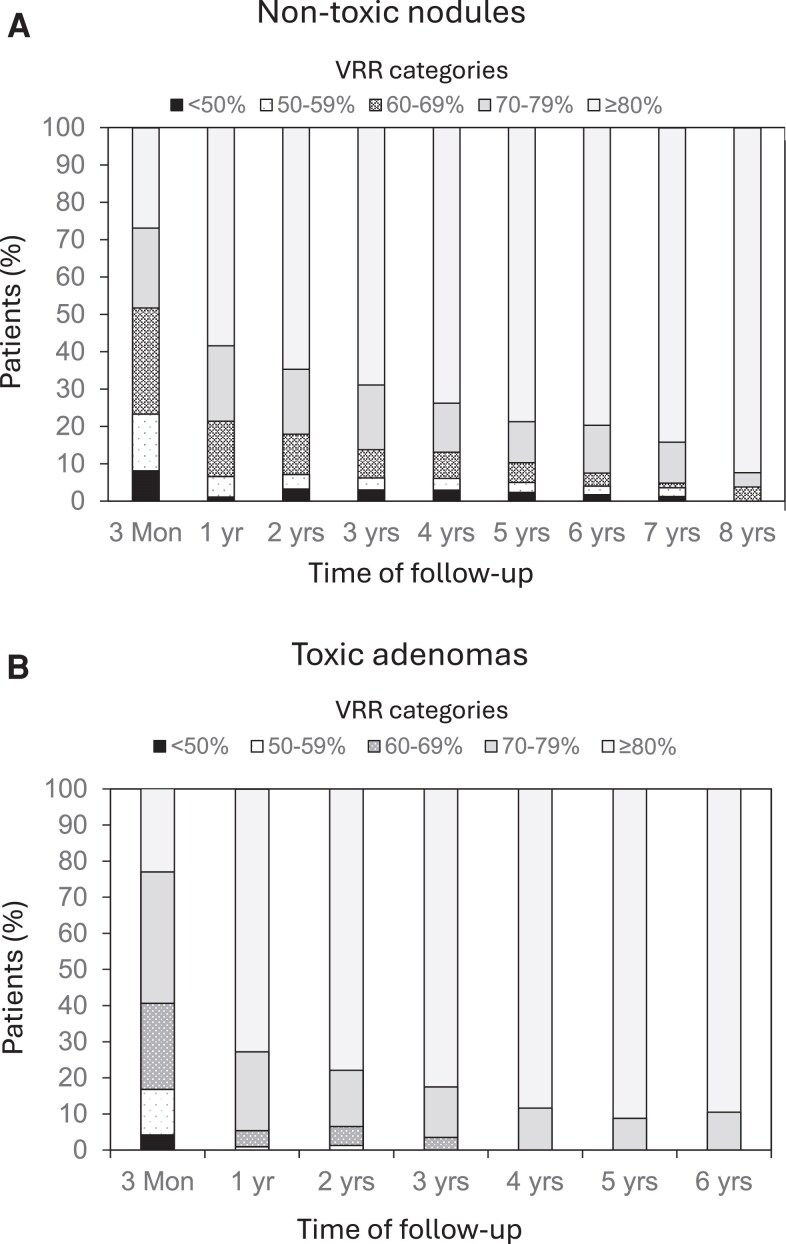
Percentage of patients in categories of nodule volume reduction (VRR) at each follow-up in nontoxic (A) and toxic adenoma (B) groups. Abbreviation: VRR, volume reduction rate.

The median follow-up period was 3.0 years (IQR 1-5; range 0.3-8) for the nontoxic group and 3.0 years (IQR 1-5; range 0.3-7) for the toxic adenoma group. Changes in nodule volume and VRR are presented in Table 2, categorized by different baseline nodule volume categories for both groups. Figure 2 illustrates the long-term changes in VRR categories for the 2 cohorts.

**Table 2.** Development of nodule volume reduction according to categories of baseline nodule size and nodule function

	< 10 mL			10- < 20 mL			≥ 20 mL		
	Nodules (n)	mL	VRR (%)	Nodules (n)	mL	VRR (%)	Nodules (n)	mL	VRR (%)
Nontoxic nodules									
Baseline	440	5.5 (3.0-7.9)		226	13.5 (12.0-16.5)		217	33.7 (25.0-50.3)	
3 months	440	1.3 (0.6-2.3)	71.5 ± 14.2	226	4.0 (3.0-5.9)	68.0 ± 14.3	217	11.5 (6.9-18.7)	65.5 ± 16.2
1 yr	365	0.7 (0.2-1.4)	82.6 ± 16.2	187	2.7 (1.5-4.5)	78.1 ± 13.7	181	7.6 (3.9-13.3)	75.5 ± 14.1
2 yrs	250	0.6 (0.2-1.2)	84.3 ± 18.3	130	2.6 (1.1-4.6)	78.6 ± 16.0	129	6.9 (3.6-11.0)	76.5 ± 16.8
3 yrs	222	0.5 (0.1-1.0)	85.7 ± 15.1	112	2.2 (0.9-4.1)	80.5 ± 15.9	103	6.2 (2.9-10.2)	79.0 ± 16.6
4 yrs	188	0.4 (0.1-1.0)	87.4 ± 12.3	88	2.1 (0.8-3.4)	80.8 ± 18.4	70	5.3 (2.2-8.7)	78.8 ± 21.9
5 yrs	140	0.3 (0.1-0.8)	88.9 ± 11.2	68	1.4 (0.4-2.9)	85.4 ± 16.7	55	5.7 (2.9-9.0)	80.4 ± 16.3
6 yrs	97	0.3 (0-0.8)	89.2 ± 12.3	39	1.2 (0.4-2.3)	88.1 ± 11.8	36	4.7 (2.5-7.4)	82.6 ± 12.1
7 yrs	45	0.1 (0-0.5)	91.5 ± 11.6	20	1.0 (0.3-1.3)	91.2 ± 7.9	17	5.7 (2.8-6.8)	83.5 ± 10.4
8 yrs	13	0.2 (0-0.4)	92.6 ± 10.1	9	0.2 (0.2-0.9)	95.1 ± 4.6	4	4.3 (2.6-8.4)	83.2 ± 8.5
Toxic nodules Baseline	90	4.8 (3.2-6.6)		30	13.7 (11.0-16.0)		24	31.6 (25.0-50.0)	
3 months	90	1.2 (0.6-1.7)	70.2 ± 13.9	30	3.6 (3.0-4.7)	70.3 ± 8.6	24	8.8 (6.5-13.7)	69.4 ± 12.5
1 yr	70	0.6 (0.2-1.0)	85.3 ± 9.5	20	1.9 (1.2-3.1)	82.9 ± 7.4	20	4.9 (4.0-7.2)	82.1 ± 8.8
2 yrs	53	0.5 (0.1-1.0)	87.3 ± 9.4	12	2.5 (1.0-3.1)	81.1 ± 10.1	13	4.2 (2.4-6.4)	87.3 ± 7.5
3 yrs	39	0.3 (0-0.9)	89.3 ± 9.4	10	2.5 (0.9-3.0)	83.8 ± 8.2	9	4.0 (1.8-6.8)	87.2 ± 7.9
4 yrs	28	0.3 (0-0.6)	90.8 ± 8.0	10	2.0 (0.9-3.0)	85.2 ± 9.1	5	5.0 (2.0-6.8)	85.4 ± 6.2
5 yrs	22	0.3 (0-0.6)	90.1 ± 7.7	7	1.2 (0.3-3.0)	88.7 ± 8.9	5	5.4 (1.8-6.5)	85.6 ± 6.4
6 yrs	15	0.4 (0-0.9)	92.2 ± 7.8	2	n = 2		2	n = 2	

Mean ± SD; nodule size, median (interquartile range).

Abbreviation: VRR, volume reduction rate

In the nontoxic patient group, median cosmetic scores decreased significantly (all *P* < .001) from 3.0 (range 0-3) at baseline to 1.0 (0-3) at 3 months and 0 (0-3) at 12 months. In the toxic adenoma group, median scores fell significantly from 2.0 (0-3) to 0 (0-3) and 0 (0-2), respectively. Median symptom scores similarly decreased from 3.0 (range 0-10) to 0 at 3 months in both groups (range 0-10 for nontoxic, 0-5 for toxic adenoma), and all scores remained low throughout the observation period.

Out of 131 toxic adenoma patients, 28% were on thyrostatic medication at baseline. The other 72% had a preablation TSH level of 0.15 ± 0.14 IU/L. All patients stopped thyrostatic medication the day after RFA. At 3 months, the mean TSH level had increased to 1.1 ± 0.8 IU/L (*P* < 0.001) and stayed between 0.8 and 1.3 IU/L until the end of the observation. The energy released per milliliter nodule volume was significantly higher in the toxic adenoma group than in nontoxic nodule patients (*P* < .003); see also Table 1.

In our study of nontoxic nodules, we examined how predictors such as sex, age, initial nodule volume, percentage of cystic content, and energy output per milliliter of nodule volume affected the VRR after 12 months using multiple regression analysis (R² = 0.15, *P* < .001). The results indicated a significant negative association with the initial nodule volume (ß = −0.223, *P* < .0001) and a positive correlation with the percentage of cystic content (ß = 0.356, *P* < .001).

For toxic adenomas, the same model revealed a significant correlation (R^2^ = 0.06, *P* = .03), showing that only the energy per milliliter of nodule volume was positively associated with VRR at 12 months (ß = 0.28, *P* = .013).

### Regrowth and Relapse

**Figure 3.**
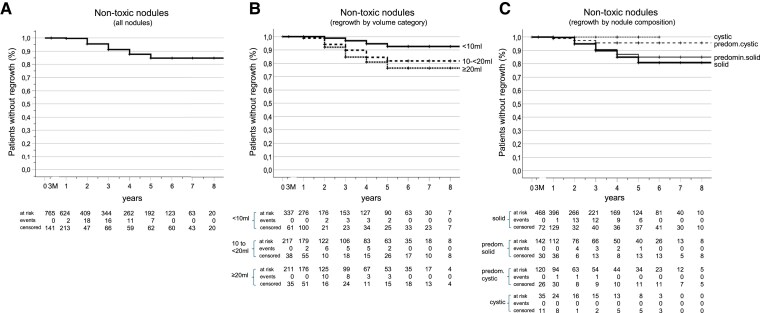
Kaplan–Meier analysis of cumulative incidence of patients with regrowth: (A) for overall cohort of patients with nontoxic nodules; (B) for patients according to different categories of baseline nodule size (univariable Cox regression analysis, model *P* < .001); (C) for patients according to different categories of baseline nodule composition (univariable Cox-regression analysis, model *P* = .08).

**Figure 4.**
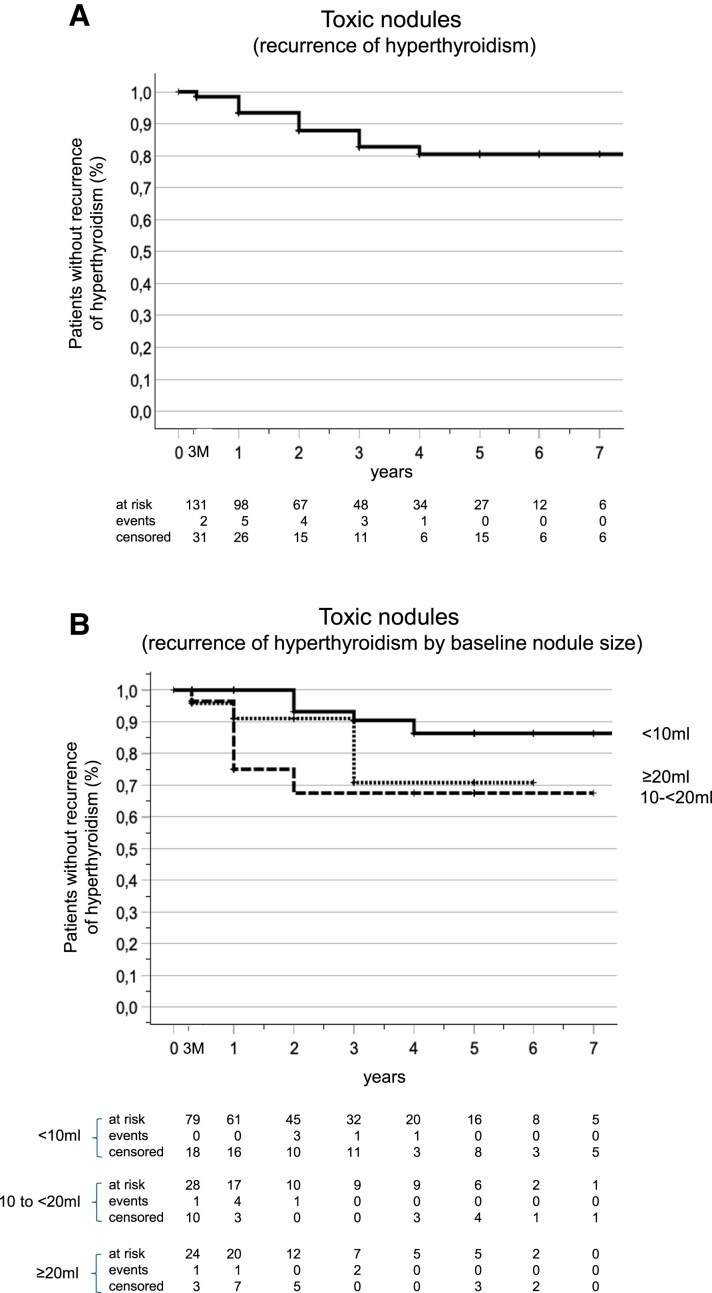
Kaplan–Meier analysis of cumulative incidence of relapse of hyperthyroidism for overall cohort of patients with toxic adenomas (A) and for patients in different categories of baseline toxic nodule volume (B) (univariable Cox regression analysis, model *P* = .01).

Table 3 and Figs. 3 and 4 present data on the number of patients experiencing regrowth or relapse of hyperthyroidism, along with annual and cumulative incidence rates. The highest regrowth rates were observed between years 2 to 5 in the nontoxic group, whereas relapse of hyperthyroidism occurred earlier, from years 1 to 3. By the conclusion of this study, the cumulative incidence of relapse was 17.3% for patients with nontoxic adenomas and 21.6% for patients with toxic adenomas.

**Table 3.** Number of patients experiencing regrowth of nontoxic nodules or relapse of hyperthyroidism in toxic nodules over time

	Baseline	3 months	1 yr	2 yrs	3 yrs	4 yrs	5 yrs	6 yrs	7 yrs	8yrs
Nontoxic nodules										
Patients, n	765	765	624	409	344	262	192	123	63	20
Nodules, n	883	883	733	509	437	346	263	172	82	26
Nodule volume, mL, median (IQR)	10.1 (5.5-20)	2.9 (1.1-6.3)	1.7 (0.6-4.1)	1.4 (0.5-4.3)	1.1 (0.3-3.4)	0.9 (0.2-2.9)	0.8 (0.1-2.5)	0.7 (0.1-2.3)	0.5 (0.1-2.0)	0.2 (0.1-1.2)
Volume reduction rate, % (mean ± SD)		69.1 ± 15.0	79.7 ± 15.4	80.9 ± 17.7	82.8 ± 15.9	84.0 ± 16.7	86.2 ± 14.2	87.6 ± 12.4	89.8 ± 10.9	92.0 ± 8.9
Patients with regrowth, n		0	2	18	16	11	7	0	0	0
Incidence, %		0	0.3	4.4	4.6	4.1	3.6	0	0	0
Cumulative		0	0.3	4.7	9.6	13.7	17.3	17.3	17.3	17.3
incidence, %										
Toxic nodules										
Patients, n	131	131	98	67	48	34	27	12	6	
Nodules, n	144	144	110	78	58	43	34	19	9	
Nodule volume, mL, median (IQR)	7.2 (4.2-14.3)	1.9 (1.0-3.9)	1.0 (0.4-2.1)	0.8 (0.3-2.1)	0.7 (0.1-2.1)	0.6 (0.1-1.8)	0.6 (0.2-1.7)	0.6 (0-1.6)	0.4 (0-0.6)	
Volume reduction rate, % (mean ± SD)		70.1 ± 12.7	84.3 ± 9.0	86.3 ± 9.4	88.0 ± 9.1	88.8 ± 8.3	89.1 ± 7.7	90.0 ± 8.7	93.7 ± 5.9	
Patients with relapse of TSH ≤ 0.4		2	5	4	3	1	0	0	0	
Incidence, %		1.5	5.1	5.9	6.2	2.9	0	0	0	
Cumulative		1.5	6.6	12.5	18.7	21.6	21.6	21.6	21.6	
incidence, %										

Patients within the nontoxic nodule group who exhibited regrowth demonstrated statistically significant differences in baseline variables compared to those without regrowth (all *P* < .003). These include nodule volume (23.4 ± 20.2 vs 16.6 ± 21.3 mL), percentage of cystic content (11.2 ± 22.1 vs 23.3 ± 33.1%), energy delivered per milliliter “net” nodule volume (0.36 ± 0.30 vs 0.64 ± 0.81 kcal/mL), and 3-month VRR (59.5 ± 23.4 vs 84.8 ± 13.4%, *P* = .003).

Uni- and multivariable Cox regression analysis (Table 4) revealed that baseline nodule volume was a stable predictor of regrowth in the nontoxic patient group. In multivariable analysis, nodule volumes of 10−<20 mL [hazard ratio (HR) = 2.71; 95% confidence interval (CI): 1.14-6.45] and ≥20 mL (HR = 3.78; 95% CI: 1.57-9.06) were significantly linked to higher regrowth risk compared to nodule volumes below 10 mL. The univariable model of nodule composition did not reach significance (*P* = .08). Such a subgroup analysis is probably underpowered; nevertheless, the Kaplan–Meier analysis is shown in Fig. 3C, as the results appear interesting in a clinical context. Higher energy per milliliter of nodule volume was associated with a reduced risk of regrowth in univariable analysis. Higher patient age reduced regrowth risk in the multivariable model: each additional year reduced risk by 4% (HR = 0.96; 95% CI: 0.94-0.99), equating to a 33% lower risk of regrowth over a decade. Sex was not a significant predictor of regrowth.

**Table 4.** Univariable and multivariable Cox regression analysis of predictors of nodule regrowth in patients with nontoxic thyroid adenomas

	Patients with non-toxic nodules			
	Univariable analysis		Multivariable analysis	
	Hazard ratio (95% CI)	*P*-value	Hazard ratio (95% CI)	*P*-value
Age	0.98 (.96-1.0)	.12	0.96 (.94-.99)	.007
Sex				
Female	1		1	
Male	1.04 (.56-1.91)	.89	1.27 (.65-2.49)	.47
Baseline nodule volume				
Model		<.001		.01
<10 mL	1		1	
10−<20 mL	3.95 (1.85-8.45)	<.001	2.71 (1.14-6.45)	.02
≥20 mL	5.39 (2.57-11.2)	<.001	3.78 (1.57-9.06)	.003
Nodule composition				
Model		.08		.11
Solid	1		1	
Predominantly solid	0.82 (.41-1.65)	.57	0.68 (.32-1.42)	.31
Predominantly cystic	0.32 (.10-1.05)	.06	0.23 (.07-.78)	.01
Cystic	0.0 (.0-4.28 × 10^225^)*^a^*	.96	0.00 (.00-8.24 × 10^251^)*^a^*	.96
kcal/mL nodule volume	0.15 (.02-.91)	.05	0.13 (.01-1.31)	.08

Abbreviation: CI, confidence interval.

^*a*^The model showed numerical instability with an extremely large confidence interval; the estimate cannot be interpreted.

In the toxic adenoma group, 9 out of 15 patients who experienced relapse (60%) showed decreases in TSH levels that coincided with nodule regrowth. In 4 out of 15 patients (27%) and in 2 out of 15 patients (13%), relapse occurred while nodules were either still shrinking or stable in volume, respectively.

Patients who relapsed in the toxic adenoma group demonstrated statistically significant differences in baseline variables compared to those who did not experience relapse (all *P* < .05). These differences included nodule volume (21.8 ± 20.9 vs 11.2 ± 12.4 mL), percentage cystic content (30.3 ± 32.4 vs 14.3 ± 21.8%), and energy delivered per milliliter of nodule volume (0.35 ± 0.19 vs 0.62 ± 0.44 kcal/mL). While the 3- and 12-month VRRs were comparable, the 24-month VRR was significantly different between the 2 groups: 77.4 ± 11.0% vs 87.6 ± 8.4% (*P* = .01).

Figure 4A presents the Kaplan–Meier analysis for the timing of hyperthyroidism relapse. In univariable Cox regression analysis, baseline nodule size predicted relapse of hyperthyroidism (model *P* = .03; Fig. 4B). Autonomous adenomas measuring 10 to less than 20 mL were associated with a significantly increased risk of hyperthyroidism relapse (HR = 4.23; 95% CI: 1.29-13.91), while nodules ≥20 mL did not reach statistical significance (HR = 3.02; 95% CI: 0.81-11.26; *P* = .10) compared to nodules smaller than 10 mL. In univariable analysis kcal/mL of nodule volume was a significant negative predictor of relapse (HR 0.04; 95% CI: 0.002-0.86; *P* = .04). Other uni- or multivariable Cox regression analyses showed no significant association with relapse risk.

### Follow-up After Regrowth

In the nontoxic group, 50% of patients with regrowth (n = 27) underwent a second RFA treatment. The baseline nodule volume for this group was 24.4 ± 17.8 mL, and the smallest mean value observed following RFA intervention was 6.7 ± 6.0 mL. Prior to the second RFA, the nodule volume measured 13.5 ± 10.6 mL and was reduced to 3.3 ± 4.9 mL, resulting in a final VRR of 85.1 ± 13.2% relative to baseline. Nine patients (16.6%) proceeded with surgical intervention; 1 patient had follicular neoplasia prior to surgery. However, histological examination confirmed that all nodules were benign. Eight patients without clinical symptoms and with nodule volumes still below baseline values opted for continued observation. One patient received radioiodine therapy, and another underwent ethanol ablation due to cystic degeneration of the nodule remnant. The status of an additional 8 patients is unknown.

In the toxic adenoma group, 11 out of 15 patients with relapse of hyperthyroidism received radioiodine treatment after a 10-day course of suppressive thyroxine or a second RFA depending on the location of the toxic nodule remnant and patient preference. The baseline nodule volume in the subgroup of radioiodine treated patients was 15.5 ± 12.6 mL, and the lowest mean nodule volume recorded was 2.4 ± 1.9 mL (VRR 83.1 ± 6.7%). Before radioiodine treatment, nodule volume had increased to 3.3 ± 1.6 mL (VRR 69.5 ± 17.1%) and changed to 1.3 ± 0.7 mL (89.6 ± 7.9%) at their last visit. The administered I^131^ dosage averaged 151 ± 59 MBq. At the time of final analysis, all these patients have been euthyroid for an average mean duration of 70.5 ± 20.7 months. Out of 4 patients with relapse of hyperthyroidism who received a second RFA, 3 have remained euthyroid for the past 1 to 3 years, and 1 patient with multifocal autonomy has relapsed again after 2 years and is now scheduled for radioiodine treatment.

### Complications

Table 5 summarizes all side effects and complications. The overall complication rate was 3.7%, with 1.1% mild, 2.3% moderate, and 0.3% severe. Temporary vocal cord paralysis typically resolved within 3 months. Capsule ruptures, occurring 2 to 5 weeks postprocedure often due to prior strain, were treated conservatively. Four of these patients developed small fistulas, with 1 requiring a small puncture incision. One case resulted in a tiny scar, all showing above-average VRRs at 12 months. There was 1 patient with what could be termed “capsule weakness,” for there were visible signs of postinterventional nodule prolapse probably due to a thermically weakened ventral thyroid capsule without sonographic indication for rupture. There was no pain, and the situation improved spontaneously. Two previously undiagnosed Graves' disease cases emerged 2 months post-RFA. One lactating patient required surgery for a wound infection.

**Table 5.** Side effects and complications during or after radiofrequency ablation of nontoxic and toxic adenomas of 896 patients

Side effects	Number (n)	Patients (%)		Number (n)	Patients (%)
Neck pain	18	2	Sore throat	3	0.3
Hematoma			Palpations	3	0.3
Subcutaneus	14	1.5	Hypotensive reaction	2	0.2
Intranodal	6	0.6	Coughing	2	0.2
Intramuscular	3	0.3	Neck swelling	1	0.1
Dysphagia	12	1.3	Vomiting	1	0.1
Subfebrile temperature	9	1.0	Increased salivation	1	0.1
Vasovagal reaction	6	0.6	Panic attack	1	0.1
Blood pressure increase	5	0.5	Exhaustion	1	0.1
Band-aid allergy	4	0.4	Depressive mood	1	0.1

Mild complications	Number (n)	Patients (%)	Moderate complications	Number (n)	Patients (%)	Severe complications	Number (n)	Patients (%)
Temporary hyperthyroidism	5	0.5	Vocal cord palsy (temporary)	12	1.3	Vocal cord palsy (permanent)	2	0.2
Temporary hypothyroidism	2	0.2	Capsule rupture	5	0.5	Wound infection (surgery)	1	0.1
Headache	2	0.2	Graves‘ disease	2	0.2			
Shoulder pain	1	0.1	Hyperthyroidism with elevated antibodies	1	0.1			
Capsule weakness	1	0.1	Deterioration of autoimmune thyreopathy	1	0.1			
			Permanent hypothyroidism	1	0.1			

Comparing the periods before (2014-2017) and after (2020-2024) the technical improvements implemented in the RFA routine between 2017 and 2019 (primarily hydrodissection and rescue maneuver), there was a significant reduction in complications when all 3 Society of Interventional Radiology categories (18.1% vs 5.7%, *P* < .05) or when only moderate and severe complications are considered (3.5% vs 1.2%, *P* < .05).

### Internet Survey

The results, which are available for 377 of 538 patients (yielding a response rate of 70%), are presented in Table 6.

**Table 6.** Internet-based anonymous survey on subjective experiences related to the RFA procedure

	Survey 1 (2018)	Survey 2 (2019)
Number of patients with responses*^a^*	252	125
**Questions**	**Responses** *^b^*	
Were you well informed before the procedure?	98	98
Was the patient information and consent form informative enough for you?	97	96
How satisfied were you with the care you received on the day of treatment?	99	99
How excited were you on RFA day?	47	50
How painful was the procedure?	23	18
What level of discomfort did you experience in the days that followed?	19	23
How satisfied are you with your check-up results?	94	93
Would you generally recommend RFA treatment based on your experience?	98	98

Abbreviation: RFA, radiofrequency ablation.

^*a*^The survey was sent to 538 patients; 377 patients responded (response rate: 70%).

^*b*^Mean values of the 100-point scale.

## Discussion

Long-term RFA studies are rare and show considerable heterogeneity mainly due to differences in treatment frequency, patient selection, and the definition of regrowth. The present study reports data collected from 2014 to 2024 in an experienced Austrian center, aiming to manage treatment of nontoxic and toxic adenomas by a single RFA intervention.

For nontoxic nodules, the most important result of our study was a cumulative regrowth rate of 17% over a maximum follow-up time of 8 years. Most of the regrowth occurred between years 2 and 5 and then stopped. A retrospective Italian multicenter study with 219 RFA patients found an almost identical regrowth rate of 20% over a 5-year observation period after a single RFA treatment with comparable baseline nodule size (20). The slightly lower VRR of 77% compared to our study (86%) can be explained by the larger proportion of solid nodules included. A further study by the same first author found a similar regrowth rate of 23% in 78 patients with nodules averaging 11 mL in size also over 5 years (21).

Another retrospective study with available data of 71 nodules at the 5-year follow-up visit (out of 215 patients at baseline) reported a nodule volume increase above baseline in 4.1% of the patients (10). It remains unclear whether this represents an incidence rate or whether further cases would have to be added based on the currently used regrowth definition.

Recently, Park et al reported regrowth in 11% of patients and a cumulative incidence of 20%, with 47% of the patients receiving more than 1 RFA treatment (456 patients at baseline and 169 after 10 years) (6). The increased number of RFA treatments likely reflects a higher proportion of solid or predominantly solid nodules and possibly larger nodule size. The authors provided a “mean” nodule volume of 21 mL in their study. The mean value in our study is 17 mL, but as the data are not normally distributed, we report the median (10.1 mL [IQR 5.5-20]), so baseline nodule size cannot be directly compared. European long-term studies typically publish single RFA treatment results (10, 20-22), whereas Asian studies often describe outcomes from multiple RFA interventions (2-4, 6, 23). Cultural views may vary on the speed and completeness of achieving therapeutic goals. RFA treatments were repeated for residual vital nodule tissue, which was not done in our study. Given that the results of all 3 referenced studies (6, 20, 21) are comparable, it may be inferred that not all sonographically vital residual tissue necessarily exhibits growth potential. Frequently, a vital nodule portion is encircled by nonviable tissue, suggesting that, in certain cases, restricted blood flow or other surrounding factors may limit further growth. Nodule selection is certainly crucial, and unfavorable nodule locations could more often constitute a contraindication for RFA intervention in European cohorts.

Initial nodule size was the main predictor of regrowth in our study. Nodules measuring 10 to <20 mL and ≥20 mL increased regrowth risk by 2.7 and 3.8 times, respectively, compared to those <10 mL. Park et al (6) found a similar hazard ratio of 2.3 for nodules ≥20 mL, while Bernardi et al noted a significant yet smaller increase (HR of 1.18 and 1.40 for an increase in baseline nodule volume of 10 and 20 mL, respectively) (20).

Our multivariable Cox regression analysis found that older age (a 33% risk reduction per decade) and predominantly cystic nodules are linked to lower recurrence risk. Bernardi et al reported similar significant findings regarding age and partially for nodule composition (20), while Park et al found no significant predictors of nodule regrowth other than nodule size, which could be attributed to the frequency of RFA treatments in their study (6).

For toxic adenomas, the remission rate in our study was 78% over a maximum of 7 years, with most functional recurrences occurring after 1 to 3 years. A meta-analysis and literature review reveal that 50% of patients with toxic adenomas achieve remission (24) and 59% show a therapeutic response (25) after 1 RFA treatment for toxic adenomas. On average, studies included in the meta-analysis comprised 23 ± 10 patients (range 3-41), with 11 out of 13 studies lasting only 1 year. The largest study by Mauri et al (22) is a multicenter study involving 118 patients after single RFA treatment of autonomous adenomas (median nodule volume: 9.9 mL), showing clinical success in 57% of patients at 3 years, favoring small nodules. In a comparative meta-analysis, the superiority of radioiodine therapy over RFA treatment therefore appears to be justified (24). Explaining why our remission rate exceeds the meta-analysis calculations is challenging, but a larger number of interventions and increasing experience of 1 interventionist over time appear likely. Whereas in our study any thyrostatic medication was withdrawn already the day following RFA, 40% of patients in the study by Mauri et al (22) were still on such medications 1 year after the intervention. In our study, the requirement for thyrostatic medication after intervention was considered a relapse and censored accordingly. The energy delivered per milliliter of toxic adenoma volume needs to be significantly higher than for nontoxic nodules, and advanced techniques to prevent regrowth or relapse should be applied. In our study, in 14 out of 15 patients, recurrence of hyperthyroidism was managed with either low-dose radioiodine therapy (n = 11) or a second RFA (n = 3). Ultimately, 130 of the initial 131 patients (99.2%) were euthyroid at their last follow-up without the need for thyroxine replacement therapy. If these study results are validated by other groups, such a treatment strategy may offer a lower risk of subsequent hypothyroidism compared to a prevalence of approximately 30% after lobectomy (26, 27) or radioiodine treatment (28, 29).

Symptom score improvement and patient satisfaction after the RFA procedure are well documented (30), but it remains interesting to gather responses on other subjective experiences related to the procedure. In our study, the response profile was nearly identical in 2 separate surveys: notable anxiety before the procedure, manageable pain during and after RFA, and a high level of satisfaction with the first results. It is not feasible to quantify the value of an absent scar or unnecessary medication following RFA. Therefore, direct comparisons between operated and nonoperated patients are only partially possible.

Overall, our complication rate was comparable to that of published meta-analyses (31, 32). Temporary recurrent paresis in our study occurred in 1.3% of cases, which is within expected limits. Some instances might have been prevented with the hydrodissection maneuver, which was introduced later. Capsular ruptures had an incidence of 0.5%, aligning with published ranges (0.2-2.5%) (33). These ruptures often require prolonged treatment and patience on the part of patients.

This retrospective analysis has limitations, primarily selection bias due to patient loss during follow-up. Satisfied patients might skip further check-ups, while those with nodule regrowth or hyperthyroidism relapse may consult other doctors or delay treatment. However, patients who dropped out showed no statistically significant differences in baseline nodule volume or VRR during follow-up compared to those who remained. Selection bias in our study mainly arises from nonstandardized nodule selection for RFA but is less significant once treatment has been initiated since all available follow-up data were included, with only a few mentioned justifiable exclusions. Future studies should stratify nodules by volume and location, successful hydrodissection, and other factors affecting outcomes. Our study likely has a temporal selection bias due to evolving expertise and advanced treatment techniques introduced over time. Patients with relapsed hyperthyroidism were managed on a case-by-case basis without standardization. Unlike other studies, our surgery rate was only 1.1%, as most recurrent patients chose a second RFA. We have had no false-negative cytologic results, and all histologic results from the 9 operated patients were benign.

In summary, a single RFA intervention was highly effective mid to long term with an acceptable complication rate for both nontoxic and toxic autonomous adenomas in an Austrian single-center experience over a decade. Smaller solid or toxic and larger cystic, nontoxic nodules are particularly promising indications for RFA. At the final follow-up, 99% of patients with toxic adenomas were euthyroid. To reach this outcome, 11% of these patients had to undergo additional low-dose radioiodine therapy or a second RFA.

## Data Availability

Some or all datasets generated during and/or analyzed during the current study are not publicly available but are available from the corresponding author on reasonable request.
